# A Fatal Case of Uræmia

**Published:** 1862-12

**Authors:** Addison Niles

**Affiliations:** Quincy, Ill.


ARTICLE XLI.

### A FATAL CASE OF URÆMIA.

Reported by ADDISON NILES, M.D., Quincy, Ill.

Ediom Hurd, aged twenty-four years, a soldier, brought from Pittsburgh Landing to Quincy, May 12th, 1862.

From the history obtained, it appears that he suffered from diarrhœa for two weeks previous, and that his case was reported as typhoid fever.

He was greatly prostrated: the tongue brown and dry, pulse feeble with no ferbile excitement; vomitimg and purging of a dark green fluid, occurred several times during the twenty-four hours; respiration and the intellectual functions undisturbed. He had no appetite for food, and complained of fulness of the stomach. A large bed-sore was found over the sacrum, and smaller ones on each of the hips. A nutritious diet, and a supporting plan of treatment, including per sulphate of iron and quinine were advised; under which, the tongue became moist and clean, and the diarrhœa considerably diminished, and the strength improved.

The first of June, a swelling appeared below and in the front of the left ear, and patches of diphtheritic membrane were observed in the mouth, with soreness of the throat and cough; the swelling disappeared by suppuration.

The urine was now examined, and found abuminous, specific gravity 1·010. By the microscope, blood disks, granular epithelical casts, and renal epithelium were observed. The blood disks increased until his death, before which time the fluid assumed a red appearance. Three days before his death, the vomiting and purging ceased, and the diphtheritic patches disappeared under a lotion of nitrate of silver. From this time he began to sleep, at first he could be aroused, but the drowsiness increased, and profound coma followed, with occasional convulsions; death occurred June 25th.

In the afternoon of the same day, an examination was made by Drs. ROBBINS and CHURCHMAN, practitioners of this city, and graduates of Jefferson Medical College, and myself. The mucous membranes of the stomach and bowels was inspected in every part. The surface was pale generally, and anemic, and the membrane sound and normal, excepting a portion of the colon, in which the folicles were enlarged, the surface appearing as though fine sand had been sprinkled over it. The peritoneum, lungs, heart, and liver and spleen appeared normal. An old adhesion was found between the two surfaces of the pleura on the left side. One ounce of serum was taken from the pericardium, and four ounces from within the cranium, chiefly from the ventricles of the brain, which otherwise appeared healthy. The right kidney was much engorged, and the left had a cyst developed in it.

From the fluid in the brain twenty-five grains of urea were precipitated by nitrate of mercury, after the process of LIEBIG. The kidneys were examined by the microscope, and the convoluted tubes found generally diseased. Some portions were stripped of their epithelial lining, and in some, small cells were developed in its place; others were filled with blood disks and fragments of epithelium. The immediate cause of death, doubtless, was the effusion in the brain, obstructing its action. This resulted from the urea, and, probably, other renal matters retained in the blood, in consequence of the failure of the kidney from disease to eliminate them.

It is worthy of remark that so long as the vomiting and purging continued, the head did not suffer. Prof. HAMMOND has shown by injecting urea into the veins of dogs deprived of kidneys, that vomiting and purging prolonged life by expelling the poison. The *post mortem* appearances resemble those found by Dr. ALONZO J. PHELPS in the camp near Corinth. By his report in the August number of the *American Medical Monthly*, it does not appear that the kidney or its secretion was examined by the microscope. The doctor justly concludes that the disease "is not dothinenteritis," or typhoid fever proper.

